# Isolation, Characterization, and In Vitro Cell Studies of Plant-Based Exosome-like Nanovesicles for Treatment of Early Osteoarthritis

**DOI:** 10.3390/ijms26052211

**Published:** 2025-02-28

**Authors:** Narjes Rashidi, Chaozong Liu, Pascale V. Guillot, Maryam Tamaddon

**Affiliations:** 1 Institute of Orthopaedic & Musculoskeletal Science, Division of Surgery & Interventional Science, Royal National Orthopaedic Hospital, University College London, London HA7 4LP, UK; narjes_rashidi@yahoo.com (N.R.); chaozong.liu@ucl.ac.uk (C.L.); 2 Research Department of Maternal and Fetal Medicine, Elizabeth Garrett Anderson Institute for Women’s Health, University College London, London WC1E 6DB, UK; p.guillot@ucl.ac.uk

**Keywords:** plant-based exosome therapy, bioactive nanovesicles, early osteoarthritis treatment, therapeutic plant-based nanoparticles, grapefruit exosome-like nanovesicles

## Abstract

Osteoarthritis, affecting over 8 million people in the UK, remains a debilitating condition with limited treatment options. Current therapies primarily address symptoms and can exacerbate joint damage over time. Developing disease-modifying drugs that alleviate inflammation and promote joint regeneration is crucial for long-term patient benefit. This study investigates the potential of exosome-like nano-vesicles isolated from grapefruit juice (GEVs) as a novel therapeutic approach for osteoarthritis. GEVs possess regenerative properties and present a promising avenue for clinical translation. In this study, nano-vesicles were isolated and characterized in terms of protein quantification, size, and morphology. In vitro studies demonstrated the safety and efficacy of GEVs, showing an enhancement in human chondrocyte migratory activity of over 13%. GEVs exhibited a dual mechanism of action, reducing inflammation and oxidative stress while promoting cellular regeneration. Specifically, they reduced the expression of COX2 and PTGS2, markers associated with inflammation and pain sensitization, and enhanced the expression of antioxidant genes SD2 and GPX in osteoarthritic-like chondrocytes. Additionally, GEVs downregulated the expression of ADAMTS-5 and hypertrophic COL10 while upregulating chondrogenic markers ACAN, COL2, and SOX9. This research signifies a significant advancement in osteoarthritis therapy, offering a natural, safe, and cost-effective treatment option with the potential for long-lasting benefits. Clinical translation of GEV therapy holds promise for improving patient outcomes and reducing the burden on healthcare systems.

## 1. Introduction

Osteoarthritis (OA) is one of the most prevalent joint diseases that affects more than 8 million people in the UK and 240 million worldwide [1]. Patients with OA suffer from joint pain, stiffness, reduced mobility, and diminished quality of life [2]. Currently, treatments are mainly palliative, which primarily focus on symptom management but do not contribute to the regeneration of the joint. Several key factors contribute to the incidence of OA, such as occupation, sports participation, musculoskeletal injuries, obesity, and gender [3]. While current treatments like corticosteroid injections, viscosupplementation, and even surgical interventions such as joint replacement offer some relief, they primarily focus on symptom management and do not effectively repair damaged cartilage or reverse disease progression [4]. As a result, the need for regenerative therapies that address the root cause of OA remains critical. These therapies, aiming not only to alleviate symptoms but also to ‘renew’ affected joints, often involve the use of stem cells or their products, such as extracellular vesicles (EVs), which offer the benefits of stem cells without associated safety concerns. EVs are lipid bilayers containing proteins and nucleic acids that are secreted by cells into the extracellular spaces and hold great promise for regenerative medicine [5]. While research has explored various EV sources like mesenchymal [6], synovial [7], and embryonic stem cells [6] for OA treatment, challenges related to delivery, safety, toxicity, and production costs have hindered clinical translation [8]. Plant-derived EVs (Plant-EVs) represent a novel category of regenerative biologics. Similar to mammalians, plant cells also export protein, lipids, and RNAs including mRNAs, microRNAs (miRNAs), and other non-coding RNAs into the extracellular space inside nanosized extracellular vesicles [9,10,11]. These plant-EVs play an important role in the developmental and defense mechanisms of plants [7]. In addition, they are shown to transfer information across kingdoms [11,12]. Plant-EVs have several advantages over their mammalian counterparts: they do not contain zoonotic or human pathogens; they are environmentally friendly, cost-effective to manufacture, and potentially an easier route to the clinic. Moreover, their edible nature allows for diverse administration routes, including injectable, transdermal, or oral routes. In addition, they are also advantageous as drug delivery tools compared to artificial nanoparticles. Finally, they have a slower clearing rate from the body, have no detectable toxicity or immunogenicity, and also display more efficient cellular uptake [13]. Although the secretion and role of EVs are less understood in plants than in mammals [14], this has opened a new avenue for exploring the interspecies communication role of plant-EVs for therapeutics and drug delivery applications. Studies on plant-EVs so far have shown their immunomodulatory/anti-inflammatory [8,15,16,17], anti-tumor [18,19,20], regenerative [18,21,22], and hepatoprotective [23] properties. Several studies have explored the effects of plant-EVs on gastrointestinal homeostasis. For instance, one study showed that vesicles from grapes targeted intestinal stem cells and induced tissue remodeling and protection against DSS-induced colitis [21]. A study conducted on ginger-EVs showed that oral administration reduced pro-inflammatory cytokines (TNF-α, IL-6, and IL-1β) and increased anti-inflammatory cytokines (IL-10 and IL-22) in colitis mice models [23]. A similar effect was seen in EVs isolated from grapefruit which showed anti-inflammatory properties by upregulating the expression of heme oxygenase-1 (HO-1) and inhibiting the production of IL-1β and TNF-α in intestinal macrophages [16].

Although plant-EVs have been explored in several disease conditions, their application in musculoskeletal diseases and joint conditions such as OA is not explored yet. In principle, since plant-EVs contain nucleic acid, including miRNA, we can expect that they participate in post-transcriptional gene regulation in the target cell [24]. In fact, several studies have suggested the prospect of cross-kingdom gene regulation by plant-derived miRNAs [24,25,26]. On the other hand, it is known that miRNAs can regulate chondrocyte (cartilage cells) signaling pathways, apoptosis, and proteinase gene expression [27]. Therefore, it is feasible that plant miRNAs can mediate chondrocyte response. Another potential effector within EVs is different enzymes that are generally associated with the protein cargo of the vesicles [14]. These proteins and enzymes are protected by the lipid bilayer and hence are shown to survive degradation and reach the target cell in their active form. In the context of OA, enzymatic antioxidants present in plant-EVs could have huge potential as they can protect the cells from oxidative damage caused by reactive oxygen species (ROS) [14]. A recent study showed that EVs from strawberries can prevent oxidative stress in human mesenchymal stromal cells, possibly due to the presence of vitamin C inside the nanovesicle membrane [28].

The present study investigated the impact of plant-EVs in modifying catabolic factors in OA-like conditions. We developed and optimized a prototype for the isolation, purification, and preservation of EVs from grapefruit (GEVs) using a modified size exclusion chromatography technique. Approximately 10^9^ highly purified GEVs were isolated from 30 mL of fruit juice, with particle sizes ranging from 120 to 170 nm. Characterization studies confirmed their morphology and protein content, and subsequent experiments demonstrated that GEVs can be internalized by chondrocytes without compromising viability. Treatment with GEVs improved cell viability, counteracting the effects of IL-1β, and enhanced chondrocyte migration, beneficial in cartilage repair. In an in vitro osteoarthritis (OA) model, GEV treatment of IL-1β-induced chondrocytes significantly modulated gene expression: upregulating antioxidant (SOD2, GPX) and chondrogenic (SOX9, COL2, ACAN) genes, and downregulating inflammatory (COX-2, PTGS2) and catabolic (ADAMTS-5, COL10) markers. These findings suggest that GEVs may reduce oxidative stress, inflammation, and catabolic activity while promoting tissue regeneration. The results highlight the potential of GEVs as a minimally invasive therapeutic for early-stage OA, offering a promising strategy for enhancing the endogenous antioxidant defense and regenerative capacity of chondrocytes.

## 2. Results

### 2.1. Protein Quantification (BCA) and Nanoparticle Tracking Analysis (NTA)

**Figure 1:**
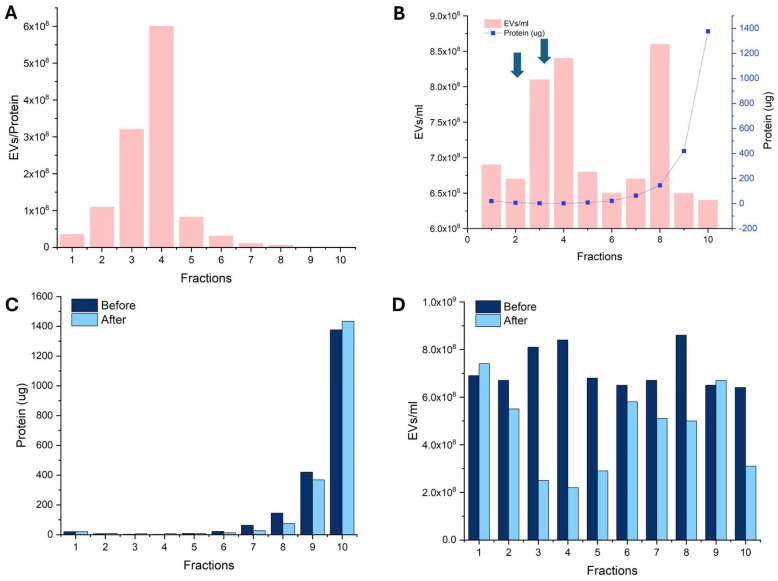
(**A**) A graphical representation of protein concentration obtained from Bicinchoninic Acid (BCA) Protein Assay analysis performed on fractions from a 35-nanometer SEC column. (**B**) Combined NTA and BCA results; fractions 3 and 4 as shown by arrows were identified as having the highest EV purity. (**C**) The effect of freeze drying on protein concentration. (**D**) The effect of freeze drying on EV concentration.

Figure 1A provides a graphical representation of the protein concentration distribution across the corresponding fractions on the 35-nanometer SEC column. The bicinchoninic acid (BCA) protein assay analysis allows us to determine the protein concentration within each sample, thereby identifying which fractions have the highest concentration of EVs. Figure 1B combines the results from nanoparticle tracking analysis (NTA) and BCA to identify the optimal fractions with the highest predicted EV content. Ideally, these fractions would exhibit the highest NTA concentration values and the lowest BCA concentration values. The fractions with the highest purity of EVs (characterized by the highest EV number and lowest protein content) were identified to be fractions 3 and 4, as indicated by the arrow in Figure 1B. To confirm consistency, three batches were tested, all showing that fractions 3 and 4 consistently demonstrated the highest EVs purity. The freeze-drying process was investigated as a method for room-temperature storage. The protein and EV concentrations before and after freeze-drying showed no significant change in protein content, with a recovery of 26–30% of the EV concentration in the pure EV fractions (Figure 1C,D).

### 2.2. Transmission Electron Microscopy

**Figure 2:**
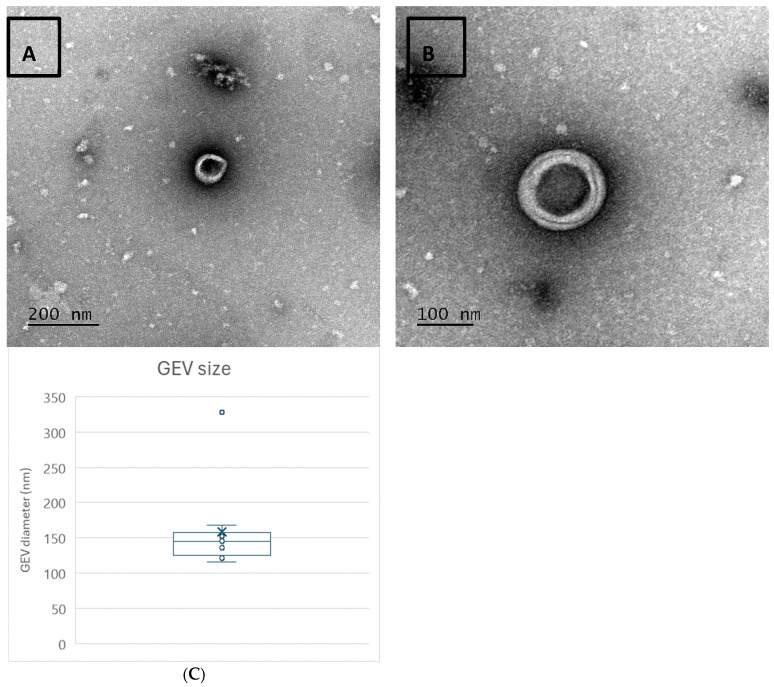
TEM images of the EVs: (**A**) signature cup-shaped morphology; (**B**) lipid bilayer structure of the EVs. (**C**) EV particle diameters (n = 11) measured using ImageJ software from their TEM images.

TEM was used to visualize EVs and characterize their morphology and size. Morphological investigation shows that EV integrity and morphology were not altered by the UF-SEC and freeze-drying processes (Figure 2A,B). Specifically, EVs appear to maintain their typical cup-shaped morphology with their phospholipid bilayer (Figure 2B). Furthermore, all EVs are in the size range of exosomes (30–150 nm) (Figure 2C).

### 2.3. Dosing

**Figure 3:**
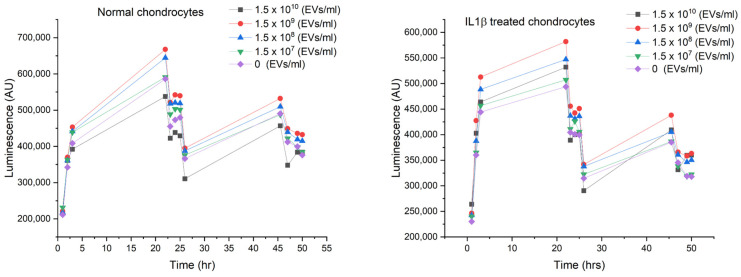
The effects of different EV dosages on healthy and IL-1β-induced chondrocytes over 50 h. The beneficial effects of EVs at concentrations of 10^8^–10^9^ particles per mL on IL-1β-induced apoptosis.

Figure 3 shows the effect of different EV dosages/populations on healthy and IL-1β-induced chondrocytes over 50 h. The RealTime-Glo™ MT cell viability assay indicates that EVs at concentrations of 10^8^–10^9^ particles per mL effectively reduce IL-1β-induced apoptosis.

### 2.4. Cell Viability

**Figure 4:**
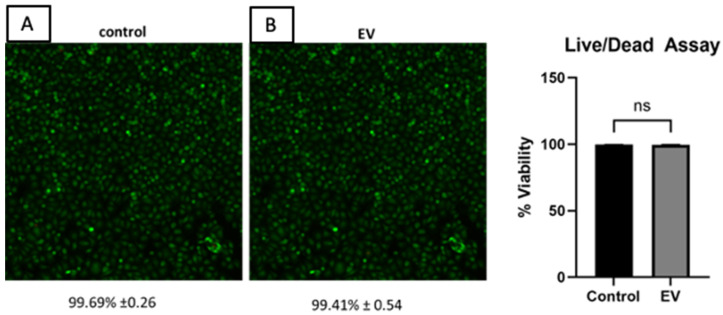
Fluorescence imaging of stained cells in a live-dead assay: green indicates live cells, and red indicates dead cells. (**A**) Live cells of healthy control human chondrocytes. (**B**) Live cells of healthy human chondrocytes treated with a very high dose of EVs. Cell density: 100,000 cells per well. No apoptosis was observed. ns = not statistically significant.

A cell viability test (Live/Dead staining) was conducted to analyze the effect of EVs on human chondrocyte viability. Figure 4 represents fluorescence images of stained cells. Chondrocytes treated with a high dose of EVs (10^8^–10^9^ particles per mL) exhibited over 99% viability, similar to the untreated control cells.

### 2.5. Cellular Uptake

**Figure 5:**
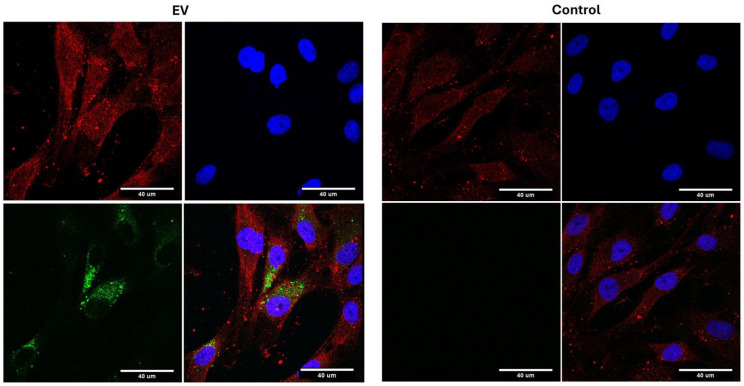
Cellular uptake of EVs. EVs are shown as green, b-actin as red, and the cell nucleus as blue. The control was incubated with the fluorescent dye alone and showed no uptake. Green: EVs; blue: DAPI (nucleus); red: b-actin.

The fluorescently labeled EVs (PKH67) were incubated with human chondrocytes overnight and processed for immunohistology. Confocal imaging (Figure 5) shows that the EVs (green) are taken up into the cell body (red).

### 2.6. Effect of EVs on Cell Migration

**Figure 6:**
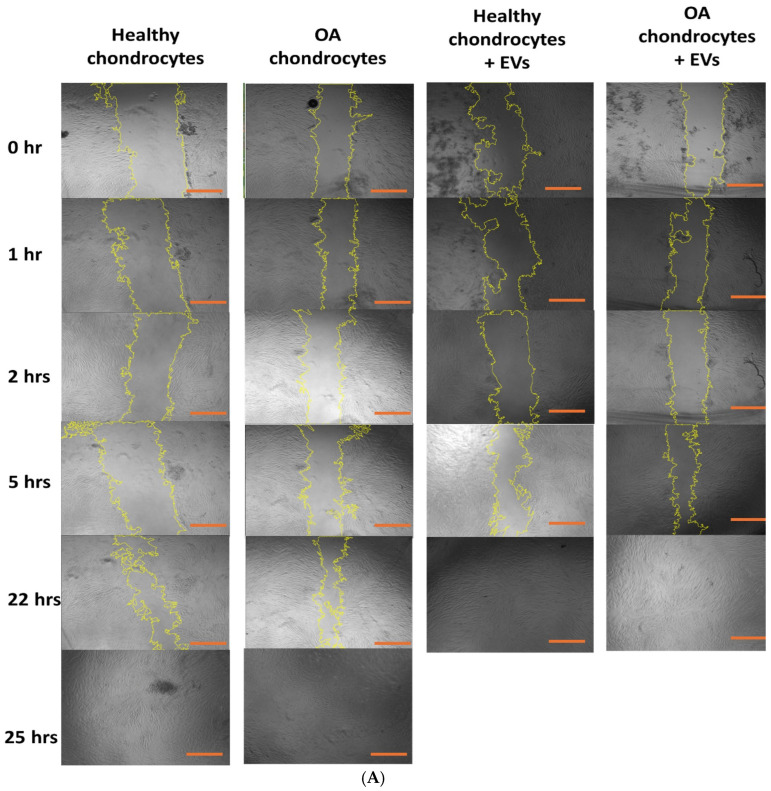

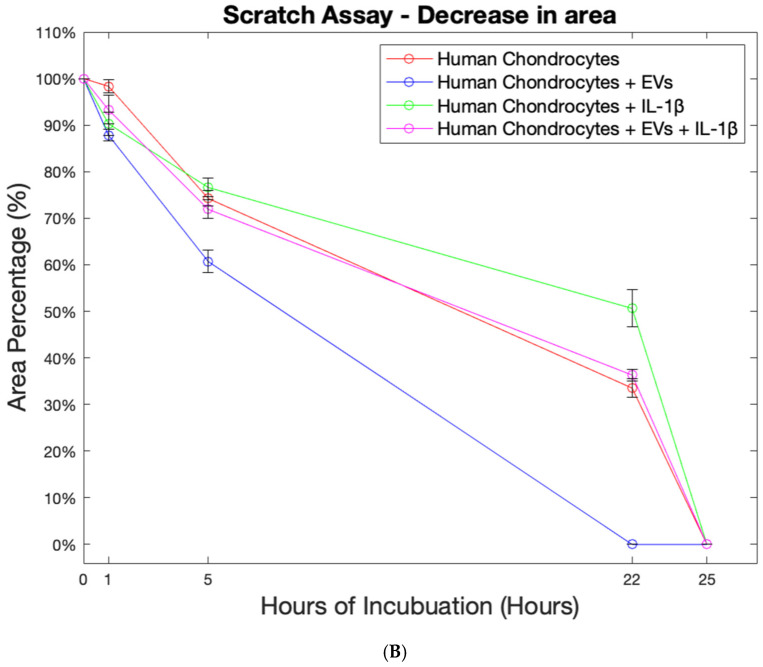
(**A**) Scratch assay showing the timelapse microscopy images of cell migration. OA chondrocytes treated with EVs were able to close the gap in less than 22 h, showing a significant effect of EVs on cell migration activity. Scale bar (red line) = 300 µm (**B**) A graphical representation of the decrease in area of the created scratch for the scratch assay results over an incubation span of 25 h.

The addition of EVs to both healthy and osteoarthritic (OA)-like chondrocytes led to increased migratory activity in cells, as shown by the wound healing assay (Figure 6A). Normally, due to the dense matrix of cartilage, chondrocytes do not migrate readily. However, increased migration is beneficial when there is cartilage injury, as the poor diffusion of cells often leads to graft failure in clinical settings [1]. Adding EVs to the cells appears to significantly facilitate their migration. While it took 25 h for untreated cells to close the scratch in the scratch assay, the EV-treated cells were able to close the gap in less than 22 h.

The decrease in scratch area becomes evident as the incubation time increases. On average, the percentage of the scratch area significantly decreased. The addition of EVs to human chondrocytes accelerated the closure of the scratch and enhanced the rate of cell migration. With EV treatment, the scratch closed within 5 to 22 h of incubation, whereas untreated human chondrocytes took approximately 25 h to fully heal. Specifically, the untreated chondrocytes reduced the scratch area from 74.25% at 5 h to 33.50% at 22 h, finally closing it completely at 25 h. In contrast, chondrocytes treated with EVs showed a reduction from 60.69% at 5 h to complete closure by 22 h (Figure 6B). This reduction in the scratch area due to the addition of EVs is significant, with an enhancement of approximately 13.6% in the rate of scratch closure. The 13.6% enhancement is derived from the difference in the rate of area reduction at 5 h (74.25% for untreated vs. 60.69% for treated), which represents the initial difference in scratch area reduction rates.

### 2.7. Gene Expression

**Figure 7:**
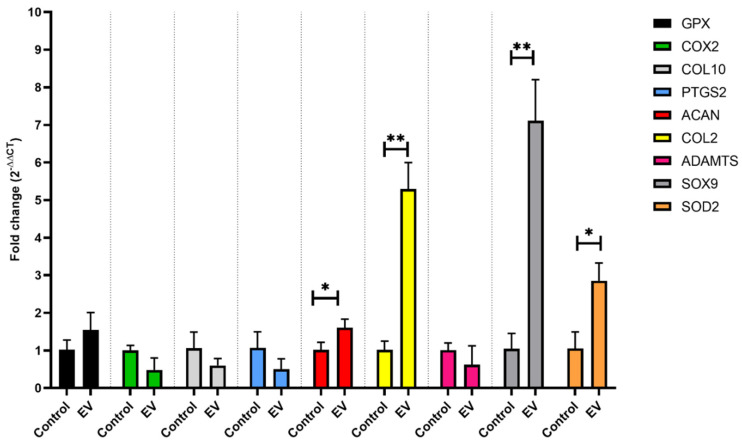
Fold change in expression of antioxidant (GPX, SOD2), inflammatory (COX2, PTGS2), chondrogenic (SOX9, COL2, ACAN), and catabolic/hypertrophic (ADAMTS5, COL10) markers in EV-treated OA cells compared to OA cells. * *p* < 0.05, ** *p* < 0.01, significant upregulation of ACAN (*p* = 0.03), COL2 (*p* = 0.0015), SOX9 (*p* = 0.004), and SOD2 (*p* = 0.01).

OA-like human chondrocytes were incubated with EVs for two days. The expressions of several genes were quantified using qPCR, including antioxidant (GPX, SOD2), inflammatory (COX2, PTGS2), chondrogenic (SOX9, COL2, ACAN), and catabolic/hypertrophic (ADAMTS5, COL10) markers as shown in Figure 7. The results revealed significant upregulation of ACAN (*p* = 0.03), COL2 (*p* = 0.0015), SOX9 (*p* = 0.004), and SOD2 (*p* = 0.01). Additionally, GPX was upregulated, while COX2, COL10, ADAMTS5, and PTGS2 were downregulated. These findings indicate a favorable modulation toward chondrogenic and anti-inflammatory states by EVs. The significant upregulation of chondrogenic markers suggests the potential regenerative effects of EVs. Moreover, the increase in antioxidant gene expression, particularly SOD2 and GPX, suggests a mechanism through which EVs may enhance the endogenous antioxidant response. Oxidative stress and elevated reactive oxygen species (ROS) levels are known contributors to OA pathogenesis [2]. Therefore, by bolstering the antioxidant defense system, EVs may effectively modulate cell behavior in affected joints.

## 3. Discussion

The main aim of this study was to explore the potential of grapefruit extracellular vesicles (GEVs) as a cell-free therapy for osteoarthritis (OA), a severe and complex joint disease. Currently, there is no permanent cure for OA, and its prevalence continues to rise. The degeneration of cartilage in diarthrotic joints leads to pain and stiffness, underscoring the urgent need for novel treatments. In this study, a prototype for the isolation, purification and preservation of GEVs was developed. The process was based on a size exclusion chromatography technique with modifications [29]. This pipeline was then optimized for efficiency, cost-effectiveness, and reproducibility, allowing for potential scalability in future clinical production. We employed a sequential isolation approach combining Size Exclusion Chromatography (SEC), ultrafiltration, and ultracentrifugation, ensuring reproducibility with consistent yields and size distribution. Given the heterogeneous nature of EVs, isolation methods significantly impact exosome quality [30]. Comparative studies show ultracentrifugation and precipitation methods yield exosomes within 30–180 nm and 30–220 nm, respectively, while ultrafiltration results in a broader 40–420 nm range [30]. Ultrafiltration recovers 1.5 times more particles than ultracentrifugation but produces a wider size distribution [31]. Our combined approach optimizes both purity and size, yielding vesicles within a well-defined 120–170 nm range.

In general, approximately 10^9^ highly purified plant-EVs could be isolated from 30 mL of starting fruit juice, with particle sizes ranging from 120 to 170 nm. After further characterization of the plant-EVs in terms of morphology and protein concentration, the interaction of purified and non-purified GEVs with healthy and OA-like chondrocytes was examined. We showed that GEVs can be internalized by chondrocytes without compromising cell viability. Moreover, the effects of various concentrations of GEVs on cell viability were assessed, revealing that GEV concentrations in the range of 10^8^ to 10^9^ particles/mL counteracted the detrimental effect of IL-1β on cell viability. Additionally, the addition of GEVs to chondrocytes increased migratory activity, particularly beneficial in the context of cartilage injury. Subsequently, a 2D in vitro cytokine model of OA was used to investigate the behavior of OA-like chondrocytes at the gene level. Chondrocytes induced with IL-1β were treated with GEVs for up to 48 h, and mRNA expressions of various genes, including antioxidant (GPX, SOD2), inflammatory (COX-2, PTGS2), chondrogenic (SOX9, COL2, ACAN), and catabolic/hypertrophic (ADAMTS-5, COL10) genes, were quantified using a real-time quantitative polymerase chain reaction.

Interestingly, treatment of OA-like chondrocytes with non-purified plant-EVs twice in 24 h reduced the expression of ADAMTS-5 (*p* = 0.005) and COX-2 (*p* = 0.02), while increasing the expression of ACAN (*p* = 0.01). Similar trends were shown when OA-like chondrocytes were treated with purified EVs. Here, significant upregulation of ACAN (*p* = 0.03), COL2 (*p* = 0.0015), SOX9 (*p* = 0.004), and SOD2 (*p* = 0.01), upregulation of GPX, and downregulation of COX-2, COL10, ADAMTS-5, and PTGS2 were observed. Degradation of aggrecans is one of the significant events in early OA, driven by matrix proteinases, mainly aggrecanases belonging to the ADAMTS family [32]. Both non-purified and purified GEVs downregulated the expression of ADAMTS-5, one of the proteinases responsible for aggrecan degradation and catabolism in a human OA [33]. Both GEV formulations also decreased mRNA expression of COX-2, the gene responsible for COX-2 isoenzyme, which is produced largely during inflammation and aids the synthesis of inflammatory prostaglandins (PTGS2) and sensitization to pain [34]. The expression of PTGS2, which is involved in collagen degradation and the inhibition of proteoglycan synthesis was also decreased. Studies show that oxidative stress, which results from an imbalance between the production of ROS and their removal by the antioxidant system, is one of the factors in the onset and pathogenesis of OA [35]. Elevated ROS levels have been observed in OA patients, contributing to synovial inflammation and subchondral sclerosis, a major factor in OA pain [36]. Evidence of heightened ROS production in OA cartilage comes from diseased chondrocytes and the detection of lipid peroxidation and nitrosylation products in synovial fluid and cartilage [37]. Directly targeting these pathways may offer therapeutic potential, but human studies on plant-derived EVs, particularly GEVs, are needed to assess their antioxidant effects under physiological conditions.

To prevent ROS-mediated damage chondrocytes produce several antioxidant enzymes including the SOD and GPX [35]. Treatment with purified EVs increased the expression of both SOD2 and GPX in OA-like chondrocytes, expressions of which are downregulated in OA joints [35,37]. Considering the increase in the expression of the antioxidant genes, this pathway could provide one potential mechanism of action. It is possible that by improving the endogenous antioxidant system, the EVs can modulate the behavior of the affected cells. Another important event in OA is the hypertrophic differentiation of chondrocytes, which is characterized by high expression of COL10 and low expression of cartilaginous specific markers, such as ACAN, COL2, and SOX9 [38]. Here, with the addition of purified EVs, COL10 expression was downregulated, while COL2, SOX9 and ACAN were significantly upregulated. This significant upregulation of chondrogenic markers shows the regenerative effects of the EVs.

The therapeutic effects of GEVs on osteoarthritic cells align with previous findings on plant EVs in other conditions. Grapefruit exosomes isolated via ultracentrifugation have demonstrated antioxidative, anti-inflammatory, and anti-cancerous effects [39], while those obtained through PEG-based precipitation have shown wound healing properties [40]. Similarly, ginger-derived exosomes isolated by sucrose gradient centrifugation exhibit anti-inflammatory and hepatoprotective effects [18,23], and garlic-derived exosomes obtained through sequential centrifugation, standard centrifugation, and PEG-based precipitation display anti-inflammatory, anti-obesity, and anti-cancerous properties [41]. Despite variations in isolation techniques and target tissues, the consistent anti-inflammatory and antioxidative effects of plant EVs suggest shared mechanisms that may be relevant for OA treatment.

Unlike plant EVs, which have not been studied in OA, human-derived exosomes have shown promise. Studies using high doses (1 × 10^10^–5 × 10^10^ particles/mL) reported significant improvements in OA models [42,43]. Our study demonstrates that GEVs, isolated via SEC and ultrafiltration, exhibit chondrogenic effects at lower concentrations, suggesting comparable or even greater efficacy.

These results allude to the striking potential of plant-EVs to be used as a treatment for OA that can improve the endogenous antioxidant system of the chondrocytes, and reduce inflammation and pain markers while having regenerative effects by improving collagen and aggrecan production. These promising results have the potential for translation as a minimally invasive and regenerative product for the early stages of OA.

### Limitations and Future Direction

While this study highlights changes in the expression of genes related to chondrogenesis in response to GEVs, the precise mechanism of action remains unclear. Specifically, the role of RNA and proteins in GEVs in driving these changes are yet to be determined. Insights from previous research on plant-EVs suggest that their bioactivity is largely influenced by their cargo composition. For instance, exosomal analysis of strawberry-derived vesicles identified small RNAs, miRNAs, and high vitamin C content [28], while ginger-derived ELNs contained lipids, proteins, and RNAs, with lipids being the key modulators of NLRP3 inhibition in macrophages [44]. Given these findings, future studies on GEVs should focus on RNA cargo characterization, proteomic and miRNA-seq analysis and its potential role in modulating signaling pathways relevant to cartilage regeneration. Understanding these molecular interactions will provide deeper insights into the therapeutic potential of GEVs in chondrogenesis.

The findings of this study are based solely on in vitro experiments, which limits their direct applicability to human biology. Compared to mammalian exosomes, research on plant-derived exosomes remains in its early stages, with significantly fewer clinical trials. A search on ClinicalTrials.gov identifies only three registered studies (one withdrawn): one on curcumin for colon cancer (recruiting, NCT01294072), and two completed trials investigating ginger for IBD (NCT04879810) and oral mucositis associated with chemoradiation in head and neck cancer (NCT01668849). To advance the therapeutic potential of GEVs, future studies should prioritize both in vivo investigations in animal models and well-designed clinical trials to assess their safety, bioavailability, and efficacy under physiological conditions.

Previous research has explored the immunomodulatory effects of plant-derived exosome-like nanoparticles both in vitro and in vivo. Yi et al. (2023) reviewed current knowledge on their role in regulating inflammatory responses and the immune microenvironment [45], demonstrating that plant exosomes can modulate the balance between pro-inflammatory and anti-inflammatory effects, thereby supporting immune homeostasis. However, data on their impact on immune responses in humans and the potential consequences of systemic administration remain limited and warrant further investigation. Nevertheless, based on existing in vitro and animal studies, plant exosomes appear to have low immunogenicity.

It is important to note that other inflammatory pathways, such as TGF-β, Wnt, and NF-κB, may also be affected. However, these pathways were not examined in this study, and future research should investigate their involvement.

## 4. Materials and Methods

### 4.1. Isolation and Purification of ELVs

The isolation and purification of grapefruit exosome-like nanovescicles (GEVs) involved the following main steps carried out sequentially: Differential centrifugation, Ultracentrifugation, Ultrafiltration, and Size Exclusion Chromatography.

Sample Preparation—Fresh fruit was thoroughly washed, air-dried, and halved for juice extraction. The juice was filtered using a metallic sieve to remove large particles, then sequentially centrifuged (Thermo Scientific™ Megafuge™ 16 Centrifuge, Osterode am Harz, Germany) at 3000× *g* and 4300× *g* for 30 min at 4 °C. The supernatant was then subjected to ultracentrifugation (Beckman Coulter, Life Sciences, Krefeld, Germany) at 16,500× *g* for 60 min at 4 °C.

Vacuum Filtration—Vacuum filtration was performed sequentially using Buchner Funnel setups with progressively finer filter papers. Filtration began with grade 4 (20–25 µm) filter paper, followed by grade 1 (11 µm), then a 0.22 µm filter paper, and was completed using a sterile 0.22 µm filter.

Ultrafiltration—The supernatant was transferred into the Vivaspin tube 20 (Sartorius Stedim, Stonehouse, Gloucestershire, UK) and spun as needed to reach the final volume of 1 mL at a maximum speed of 3000× *g* in a swing rotor or 6000× *g* in a fixed rotor.

Size Exclusion Chromatography—Size Exclusion Chromatography (SEC) was performed using a 35 nm qEV IZON column (Izon Science, Christchurch, New Zealand) following the manufacturer’s protocol. The centrifuged sample was loaded onto the column, and fractions were collected in 0.5 mL increments up to fraction 10. The collected fractions were then stored at −80 °C to preserve their stability for future analysis.

### 4.2. EVs Freeze Drying

Trehalose, a natural, non-toxic sugar widely used as a protein stabilizer and cryoprotectant was used for GEV’s freeze-drying [46]. It prevents exosome aggregation and lysis due to freezing/freeze-drying. A 50 mM Trehalose solution was prepared. GEV’s were washed with the Trehalose solution by adding 1 mL of exosomes to a Vivaspin tube and 5 mL of the Trehalose solution. The tube was spun down until the initial volume (1 mL) remained in the column, and then aliquoted into 100 μL portions in microcentrifuge tubes and frozen at −80 °C. Samples were freeze-dried in a freeze-dryer (Christ Alpha 1–2 LD plus) for at least 10 h. Once freeze-dried, they were stored in a tightly sealed box. Before use, GEVs were rehydrated in ddH_2_O by adding the original volume (100 μL) to each tube and briefly vortexed. To remove Trehalose, 100 μL of reconstituted exosomes was added to the tube plus 500 μL of filter-sterilized PBS and spun down until the initial volume (100 μL) remained in the column.

### 4.3. Physical Characterization of GEVs

#### 4.3.1. Nanoparticle Tracking Analysis (NTA) for the Measurement of GEV Size and Concentration

To perform Nanoparticle Tracking Analysis (NTA), the ZetaView PMX-200 Twin instrument (Particle Metrix, Inning am Ammersee, Germany) was used, equipped with a 520 nm laser and CMOS camera. The fraction samples taken after Size Exclusion Chromatography (SEC) were first diluted to a 10-fold dilution with sterile-filtered PBS to reduce particle numbers. One milliliter of the diluted extracellular vesicle (EV) sample was then used for analysis with the ZetaView NTA machine. Before conducting NTA on the freeze-dried samples, they were reconstituted in 100 microliters of ddH_2_O to return them to their original volume. Each tube was briefly vortexed. The samples were then loaded into the machine, which measured the particle concentration and provided the necessary characteristic information, such as particle concentration, particle number, and zeta potential. The machine was set to a laser wavelength of 520 nm with a scatter filter wavelength, and the dilution factor was set to 10. For the standard operating procedures, the NTA size distribution was measured over 2 cycles and 11 positions, while the zeta potential was assessed using 3 stationary cycles. The analysis parameters were configured with a maximum area of 1000, a minimum area of 10, and a minimum brightness of 50. Pre-acquisition parameters were set to a shutter speed of 300 ms, 30 frames per second, and a minimum sensitivity of 60 at 24 °C, with all other parameters set automatically. Data analysis was performed using ZetaNavigator software (version 8.05.16 SP2), and the particle size distribution was determined using quadratic interpolation.

#### 4.3.2. Protein Quantification

Protein quantification was conducted using the Bicinchoninic Acid (BCA) Protein Assay Kit (Sigma-Aldrich, Dorset, UK) following the instructions provided with the assay kit and absorbance was measured at 562 nm. The actual concentration of protein present in the sample was determined based on the standard curve using BSA protein standards ranging from 200 to 1000 μg/mL (20 to 100 μg of total protein).

#### 4.3.3. Transmission Electron Microscopy (TEM)

Transmission electron microscopy (TEM) (JEM 1230 TEM, JEOL Ltd., Tokyo, Japan), operating at 110 kV and equipped with an UltraScan 4000 CCD camera and a First Light Digital Camera Controller (Gatan, Pleasanton, CA, USA), was used to evaluate the size and morphology of the GEVs. A TEM grid of Formvar/Carbon on 400 Mesh Copper (AGS162-4, Agar Scientific, Essex, UK) was employed. For each grid, 2 µL of EVs resuspended in H_2_O were carefully deposited onto the Formvar-carbon-coated EM grid without glow discharge. The grids were then covered and allowed to adsorb to the membrane for 2–3 min, with slight warming under a lamp to aid evaporation. The objective was to ensure most of the drop evaporated without overdrying. Subsequently, four separate 100 µL drops of H_2_O were placed on a sheet of Parafilm. The grids (membrane side down) were transferred with fine forceps to float on top of each drop of H_2_O consecutively and left to wash for 2 min. After washing, the grid was transferred to float on a 50 µL drop of 2% uranyl acetate solution, pH 7, for 20 s. Finally, excess fluid was blotted using forceps by gently touching the edge of the grid to a piece of Whatman filter paper, leaving behind a thin film over the exosome side of the grid. After washing, the grid was then transferred to the TEM for imaging. The EVs size measurement was performed using ImageJ software (1.53s).

### 4.4. Cell Experiments

#### 4.4.1. Human Chondrocyte Culture and In Vitro Model of OA-like Chondrocyte

Human adult chondrocytes (Sigma Aldrich, Dorset, UK, 402-05A) were cultured using chondrocyte growth medium (Sigma Aldrich, Dorset, UK, 411-500), with medium changes every 3 days. For all experiments detailed, chondrocytes in monolayer culture were used between passages 3–4.

To establish an in vitro model simulating osteoarthritic (OA)-like conditions, chondrocytes were induced to express an OA-like phenotype through treatment with IL-1β (Peprotech, Rocky Hill, NJ, USA). In brief, chondrocytes were exposed to IL-1β (10 ng/mL) in the culture medium for 24 h. OA-like induction was confirmed by gene expression studies.

#### 4.4.2. Cell Viability

The LIVE/DEAD assay was conducted to evaluate cell viability using a fluorescence microscope with 4× magnification to capture images from various locations on the coverslip. The cells (100,000 cells per well) were incubated for 4 h and then stained with a solution containing 8 µM calcein AM and 20 µM Ethidium Homodimer-1 (EthD-1) in 1 mL of PBS. Images were acquired using different filters on the digital color fluorescence microscope: the Green Fluorescent Protein (GFP) filter for calcein AM staining and the Texas Red (TXRED) filter for EthD-1 staining.

#### 4.4.3. In Vitro Cellular Uptake of GEVs

Exosomes were labeled using PKH67 green fluorescent dye according to the manufacturer’s instructions (Sigma-Aldrich, Dorset, UK). Further removal of the unbound dye can be performed by using the concentrate in the size exclusion chromatography procedure detailed above. The suitable SEC fractions were washed with media twice their volume using an ultrafiltration tube with a 100 kDa MWCO. Labeled exosomes were used promptly to ensure the highest possible fluorescent intensity. The EVs were subsequently co-cultured with chondrocytes at a concentration of 10 μg/mL in a serum-free medium at 37 °C for 12 h. Chondrocytes were fixed in 4% paraformaldehyde at room temperature for 15 min, followed by permeabilization with 0.1% Triton X-100/PBS for 5 min at room temperature. Subsequently, the cells were blocked with 3% BSA in 0.01% Triton-PBS for 1 h at room temperature. The nucleus was stained with DAPI for 10 min at room temperature. Finally, the cells were observed using a laser confocal microscope (Zeiss LSM 710, Carl Zeiss Microscopy, Jena, Germany).

#### 4.4.4. Gene Expression-RT PCR

Total RNA was extracted from cells using Trizol (Life Technologies, Carlsbad, CA, US) and purified with the Direct-zol RNA Microprep Kit (Zymo Research, R2060, Irvine, CA, USA), followed by reverse transcription to generate first-strand cDNA using the High-Capacity cDNA Reverse Transcription Kit (Applied Biosystems, 4368814, Foster City, CA, USA), following the manufacturer’s protocol. Quantitative PCR was conducted using Brilliant SYBR Green II (Agilent Technologies, Santa Clara, CA, USA) on an Applied Biosystems QuantStudio 5 Real-Time PCR system (Thermo Fisher Scientific, Altrincham, UK). The primer sequences used are provided below (Table 1). The primers were designed using Primer-BLAST, validated, and shown to have efficiencies ranging from 0.90 to 1.07. Each sample underwent three independent PCR reactions, and the RT-PCR data were analyzed using the 2^−ΔΔCt^ method.

#### 4.4.5. Cell Migration Activity

Human chondrocyte migration was assessed using a wound healing assay. Human chondrocytes (200 µL at 2.91 × 10^5^ cells/well) were pipetted into eight wells of a 48-well cell culture plate (Corning Costar, Corning Inc., New York, NY, USA) and incubated for 2 h. Subsequently, 300 µL of chondrocyte growth medium (Sigma Aldrich, 411-500) was added to each well, and the plate was incubated for 3 days. Four wells were treated with 250 µL of IL-1β (Peprotech, USA) for 24 h, while the remaining four wells were treated with 125 µL of TVs and 175 µL of growth medium. Once confluence was reached, a longitudinal scratch was made through the cell monolayer using a 200 µL sterile plastic tip, creating a gap to be filled by migrating cells. The cell-free area was observed using fluorescence microscopy (Eclipse TS100, Nikon, Tokyo, Japan), with images captured at 0, 1, 2, 5, 22, and 25 h post-wounding using a camera-equipped microscope (Nikon Digital Sight DS Fi-1, Nikon, Tokyo, Japan). The wound gap closure was measured using ImageJ (11.04.00, NIH, Bethesda, MD, USA). The percentage of wound space change was calculated as:

Migration % = [(average space at time 0 h) − (average space at time 24 h, 48, 72 h)]/(average space at time 0 h) × 100%

## 5. Conclusions

Progress in osteoarthritis (OA) treatment research is crucial and ongoing. Extracellular vesicles (EVs), if isolated in a reproducible and structured manner, offer immense potential for identifying OA biomarkers and novel therapeutic approaches. With their unique bio-cargo, EVs are prime candidates for the development of both biomarkers that track disease progression and cell-free therapies. By gaining a deeper understanding of how EVs function within the joint, researchers could potentially target key pathways to prevent tissue degeneration and enhance natural tissue repair mechanisms. Developing an innovative treatment for early-stage OA would have a transformative impact in the field of orthopedics.

In this study, the methods used for grapefruit’s EV isolation, purification, characterization, and cell culture have provided valuable insights into the properties and potential therapeutic roles of GEVs. The characterization techniques confirmed that freeze-drying had no significant impact on GEVs, while protein and particle concentrations within the samples were preserved. The use of trehalose was also shown to be an effective stabilizer and preservative for GEVs.

Through cell culture experiments, we observed how GEVs interact with human chondrocytes, particularly under OA-mimicking conditions induced by IL-1β. EVs significantly enhanced the rate of scratch healing in human chondrocytes compared to controls, with high cell viability observed in EV-treated groups. GEV treatment reduced the expression of inflammatory markers COX2 and PTGS2 while enhancing antioxidant genes SOD2 and GPX. Furthermore, GEVs downregulated ADAMTS-5 and the hypertrophic marker COL10 while upregulating chondrogenic markers ACAN, COL2, and SOX9—demonstrating their regenerative potential.

While these findings are promising, further investigation is needed before large-scale therapeutic applications of GEVs can be realized. A deeper understanding of GEVs’ biology, content, and function is critical, particularly given that current evidence is based solely on in vitro studies, limiting direct applicability to human biology. To advance the therapeutic potential of GEVs, future studies should prioritize both in vivo investigations in animal models and well-designed clinical trials to assess their safety, bioavailability, and efficacy. One major challenge is the development of scalable techniques for effective GEV purification. This challenge is being addressed through the development of mega columns for processing large volumes, with recent technical advancements playing a significant role in overcoming this hurdle.

## Figures and Tables

**Table 1:** Primer sequences, designed by Primer-BLAST.

Gene	Forward	Reverse
*COL2A*	GGCAATAGCAGGTTCACGTACA	CGATAACAGTCTTGCCCCACTT
*GAPDH*	TCTCCTCTGACTTCAACAGCGAC	CCCTGTTGCTGTAGCCAAATTC
*ADAMTS-5*	GCAGAACATCGACCAACTCTACTC	CCAGCAATGCCCACCGAAC
*RPL13A*	CTTTCCTCCGCAAGCGG	GTCCGCCAGAAGATGCG
*ACAN*	ACAGATGCTTCCATCCCAGC	TCACATACCTCCTGGTCTGC
*COX-2*	CAAATTGCTGGCAGGGTTGC	AGGGCTTCAGCATAAAGCGT
*iNOS*	CTGGCAAGCCCAAGGTCTAT,	TCCCCGCAAACATAGAGGTG
*MMP13*	TCCAGTCTCTCTATGGTCCAGG	TCCAGTCTCTCTATGGTCCAGG

## Data Availability

The data presented in this study are available on request from the corresponding author.
